# A method for extracting electronic patient record data from practice management software systems used in veterinary practice

**DOI:** 10.1186/s12917-016-0861-y

**Published:** 2016-10-21

**Authors:** Julie S. Jones-Diette, Marnie L. Brennan, Malcolm Cobb, Hannah Doit, Rachel S. Dean

**Affiliations:** 1 Centre for Evidence-based Veterinary Science, School of Veterinary Medicine & Science, University of Nottingham, Nottingham, LE12 5RD UK; 2 Deputy Head of School, School of Veterinary Medicine & Science, University of Nottingham, Nottingham, LE12 5RD UK; 3 Current address: Centre for Reviews and Dissemination, University of York, York, UK

**Keywords:** Electronic patient records, Extensible mark-up language, Accuracy

## Abstract

**Background:**

Data extracted from electronic patient records (EPRs) within practice management software systems are increasingly used in veterinary research. The use of real patient data gives the potential to generate research that can readily be applied to clinical practice. The use of veterinary EPRs for research in the United Kingdom is hindered by the number of different Practice Management System (PMS) providers used by practices, as obtaining and combining data from different systems electronically can be problematic. The use of extensible mark up language (XML) to extract clinical data for research would potentially resolve the compatibility issues between systems. The aim of this study was to establish and validate a method for the extraction of small animal patient records from a veterinary PMS that could potentially be used across multiple systems. An XML schema was designed to extract clinical information from EPRs. The schema was tested and validated in a test system, and was then tested in a real small animal practice where data was extracted for 16 weeks. A 10 % sample of the extracted records was then compared to paper copies provided by the practice.

**Results:**

All 21 fields encoded by the XML schema, from all of the records in the test system, were extracted with 100 % accuracy. Over the 18 week data collection period 4946 records, from 1279 patients, were extracted from the small animal practice. The 10 % printed records checked and compared with the XML extracted records demonstrated all required data was present. No unrequired, sensitive information e.g. costs or services/products or personal client information was extracted.

**Conclusions:**

This is the first time a method for data extraction from EPRs in veterinary practice using an XML schema has been reported in the United Kingdom. This is an efficient and accurate way of extracting data which could be applied to all PMSs nationally and internationally.

**Electronic supplementary material:**

The online version of this article (doi:10.1186/s12917-016-0861-y) contains supplementary material, which is available to authorized users.

## Background

The increasing reliance on practice management systems (PMS) within veterinary practice means much of the data collected during patient encounters is captured within an electronic patient record (EPR) [1–3]. Practice management systems are now widely used with over 98 % of practices utilising them [4]. Over the last decade EPRs have been increasingly used in medicine to support human health research [5–8]. In veterinary medicine the original purpose of electronic recording systems was for clinical record keeping and billing. However, a wealth of data that could be harnessed for research, particularly data for studies informing evidence-based approaches to clinical practice across a number of PMSs, and the subsequent improvement of animal health reside in these records.

Practice management systems and EPRs have been used in a number of different ways to support veterinary research. The Veterinary Companion Animal Surveillance System (VetCompass, www.rvc.ac.uk/vetcompass) collects clinical data on veterinary consultations using a veterinary version of the SNOMED codes called VeNom Codes, which are now integrated within some veterinary PMSs, to determine prevalence, static risk factors for disease and examine treatments used for particular diseases [9–11]. The Small Animal Veterinary Surveillance Network (SAVSNET, www.savsnet.co.uk) generates surveillance data to determine prevalence and static risk factors for disease, and to examine treatments used for particular diseases from real-time questionnaires within the veterinary consultation and PMS [12]. Additionally, the Banfield Pet Hospital has developed the Banfield Applied Research and Knowledge (BARK) initiative, to capture data within their own bespoke database to support research and inform the veterinarians within their clinics and wider audiences [13–15]. Unfortunately the different methods described here utilised to obtain veterinary data are not necessarily transferrable across these PMSs due to differences in database structure. There are many different commercial PMSs on which information from veterinary primary care consultations is kept [2, 4, 16]. Therefore it is a challenge to obtain and link data from different PMSs representative of all systems currently in use within veterinary practice.

A versatile approach is vital if data is to be combined from many different veterinary practices utilising many different PMSs. One way of obtaining and amalgamating information contained within disparate PMS databases is to use Extensible Mark-Up Language (XML) [17]. This is a language tool which describes the content and component parts of a document in a plain text format, which is easily readable by humans or computers. A XML schema is essentially a list of defined data fields that identify and encode information from a large database [17, 18]. Within the veterinary field, the VetXML Consortium in the United Kingdom was established in 2006 with the aim of “improving the sharing of data through the development of an industry standard data format, in order to maximise the service provided by the veterinary profession”. They have endorsed a number of XML schemas, for example: eClaims for pet insurance, Microchip Registration service, Laboratory analysis to support the fast transfer of test results and Information extraction for benchmarking purposes, which are already in use across a number of PMS. The consortium has now endorsed the Clinical Evidence schema described in this manuscript.

The aim of this study was to establish and validate a method for the extraction of small animal patient records from a veterinary PMS that could potentially be used across multiple systems. Secondary aims were to ensure the information extracted was precise and that records could be extracted completely from patients’ EPRs.

## Methods

### XML schema development

An XML schema was developed to identify fields of interest from within the PMS software of a provider who volunteered to be involved with this project, and allow the information identified to be extracted into an XML format.

For this study, the formats of EPRs within this PMS were reviewed by the research team and the system provider to determine what clinical data fields could be utilised. Twenty one data fields were identified for inclusion in the schema (Table 1). An XML schema, *Clinical Evidence (CE) XML Schema*, was then created that included the 21 data fields of interest (Additional file 1). No financial details from the invoice fields were extracted as all treatment information (see Table 1) was extracted without associated costs.

**Table 1:** The 21 fields of the XML schema

	Name of field (description where required)
Animal fields	Practice ID (numerical)
	Animal ID (numerical)
	Species
	Breed
	Gender and Neuter Status
	Notable Conditions (e.g. allergies)
	Remarks (e.g. aggressive)
	Deceased (Yes/No)
	Dangerous (Yes/No)
	Insured (Yes/No)
	Date of Birth
	Body Weight
	Body Weight units (e.g. kg)
	Last Weight Date
	Registration Date (at the practice)
Consultation information fields	Date (of entry)
	Time (of entry)
	Entered By ID (person who entered the data-numerical identification)
	Text Entry (free text for consultation and health notes, insurance details, test results)
	Diagnosis (practice specific codes or treatments (including trade name, drug name, drug dose and length of course of treatment) and prescriptions)
	VeNom Code (from VeNom coding group)

### Extracting records within a test PMS

A test version of a veterinary PMS was created by the primary author (JJD). Eighty small animal records were added into the test system taken from paper records of patients used in previous research studies. The records were anonymised by omitting several fields, including pet name, owner name and address. Each entry was allocated an Animal ID number for reference and each entry included some clinical history. Invoice details were also added to include all treatments offered or medications prescribed to be captured upon extraction, but billing details were excluded as this information was not required. The veterinarian in the test system was recorded as the primary author (JJD) and all prescriptions or treatments added to the patients’ records were selected by JJD from an integral catalogue within the PMS.

The details entered for each patient were varied for species, age, breed, neuter status and clinical condition. The test system therefore contained 80 EPRs with at least two visit records and two invoice records recorded for each patient. This totalled 326 separate record entries within the test system.

The schema was designed to identify and extract data based on a start and end date selected by the veterinarian. In this instance the dates selected corresponded to the visit dates recorded for the patient’s records (24^th^ May–1^st^ June 2011). The extraction was ﻿then ﻿run for this selected time period within the PMS with the click of a button. The schema identified and extracted all requested data into an XML file format which was imported into an excel workbook for data checking and then transferred into an Access relational database for storage and analysis.

As the extraction method was designed for use on client owned patient data, an opt-out function was required for clients who did not want to have their pets records extracted. To validate this function, the opt-out field was selected, excluding these records from extraction, in eleven records within the PMS test system.

### Validating the extraction method within a test system

To ensure all information was extracted fully from the test system, the information extracted was compared to a paper record. The number of patient records present was compared to the number extracted and the information extracted from each field compared to those present in the paper print outs. The extracted information was also checked to ensure any data from records where the opt-out field had been selected was not included.

### Extracting records using the schema within a real veterinary practice

A working first opinion veterinary practice using the PMS was recruited to enable the integration of the XML schema into a real practice. The practice was a mixed animal first opinion practice with three veterinarians working across two clinics. The practice agreed to access of their EPRs for the purpose of research, in a manner consistent with the ethical approval of the study and with the agreement of their clients. The XML schema was integrated by the PMS provider, into the practice’s PMS.

During the data collection period, posters were placed in each of the waiting rooms of both clinics to inform all clients visiting the practice about the on-going research. The posters also informed clients that if they wished to opt out they could ask their vet to exclude the patient prior to or during the consultation. The consulting vet if requested would then select the opt-out function within the client’s patient record and the patient would be omitted from the extraction. If house or farm visits were undertaken by the veterinary surgeons they were asked to use the opt out function as it could not be guaranteed the owners would be aware of the study.

The data collection ran over two 8 week periods; January 16^th^ 2012–March 11^th^ 2012 and May 2^nd^ 2012–June 25^th^ 2012. The extraction occurred on a weekly basis (Monday to Sunday inclusive) and included data recorded on all small animal patients examined at the practice within working hours (8 am–7 pm). The senior veterinarian within the practice downloaded and sent the extracted XML file to the primary author (JJD) by password protected email on the Monday morning of the week following data collection. The collected data was then imported into an Excel spreadsheet and cleaned before transfer to a dedicated and secure relational database for storage. At the end of the study a practice meeting was held to discuss the findings and outcome of data extraction and gain feedback on the experience of the staff on being involved in this research.

### Validation of the extraction method using data from the real veterinary practice

Once the EPR extraction was complete for the first 8 week data collection period, using a random number generator, a sample of 10 % of the records were chosen to be printed out in full from the practice PMS system to ensure all of the required data was successfully extracted by the XML schema. To ensure all information was extracted fully from the test system, the information extracted was compared manually by the primary author (JJD) to the 10 % of practice paper records printed. The number of patient records present in the paper record was compared to the number extracted and the information extracted from each field compared to those present in the paper print outs. Comparison of basic data (e.g. patient signalment, free text notes, diagnosis and treatment information) was made between the printouts and the extracted data. The extracted information was also checked to ensure any data from records where the opt-out field had been selected was not included.

The study was approved by the ethics committee of the School of Veterinary Medicine and Science, The University of Nottingham. All patient data was extracted and stored in the strictest of confidence for future examination and retention by the CEVM only and no external parties had access to the data. The data extracted had no patient identifiable information as the schema was designed to exclude any client or veterinary details.

## Results

### Records extracted from the test PMS

All data were successfully extracted from the test system. All data within the 21 data fields in the XML schema were extracted fully and all data within the fields were found to be 100 % accurate with no missing information. The invoice details and all treatments were also extracted in full excluding the details that were not included in the schema relating to the cost of treatments. The records from the eleven patients where the opt out function was selected were not present in the extracted dataset.

### Validating the extraction method within a test system

All data were successfully extracted from the CEVM test system and were accepted and stored in full within the relational database. Eighty animals and 326 records were extracted in full which matched the number of records held within the test dataset and all opt out records were excluded from extraction. Validation of all extracted records using paper records for comparison found 100 % accuracy and confirmed no missing information.

### Records extracted from the PMS within a real veterinary practice

Extraction of data for the two collection periods produced a combined total of 4946 small animal patient records. A relational database was used to hold the data securely and each animal appeared only once cross referenced to their visit history which facilitated the later analysis of the information.

There were a number of records extracted which were not small animal patients including farm animals (*n* = 114) and poultry (*n* = 36) with an additional 45 blank fields found. These were excluded from further analysis (*n* = 195). All data was extracted successfully and securely transferred into the relational database. None of the clients who entered small animal consultations elected to opt out of the pilot data collection period.

### Validation of the extraction method using data from the real veterinary practice

Two hundred and fifty two (10 % random sample of all extracted records in period one *n* = 2519) printed records from the practice were compared to the extracted data by the primary author (JJD). The extraction was found to have 100 % accuracy by direct visual comparison. All free text was extracted in full as was the diagnosis field, invoicing information, prescriptions and all recorded treatment given to the patient. No billing information or financial details were extracted.

### Data aggregation and analysis

The records extracted for small animals were composed of 2246 visit notes and 2700 invoices (*n* = 4946 records). The 2246 visit notes included information recorded as consultation (*n* = 1858), test results/lab reports (*n* = 292), insurance details (*n* = 64), previous history (*n* = 22), referral notes (*n* = 6) and follow up appointments (*n* = 4). Diagnostic codes (a mixture of practice and VeNom codes) were recorded for 137/2246 (6 %) visits by one veterinarian. Of the 1858 notes recorded as a consultation, 1624 were recorded simply as ‘consultation’, 83 as ‘vaccination’, and 14 as ‘phone calls’ or ‘phone consultation’.

The 4946 records contained information on 1279 patients. In the first 8 weeks of data collection, 2519 patient consultation records were extracted and invoice information for 775 patients. In the second 8 weeks of data collection, 2427 patient consultation records were extracted and invoice information for 822 patients. This produced a dataset of 1597 combined patient records; as some animals visited the practice during both collection periods, the exact number of individual animals examined over both data collection periods combined was 1279 animals (Table 2).

**Table 2:** Total number of small animals for which patient records were extracted from the real veterinary practice during each period (and combined period) of data collection

Species	First Collection Period *N*	Proportion (%)	Second Collection Period *N*	Proportion (%)	Combined Collection Period *N*	Proportion (%)
Dogs	439	57	469	57	693	54
Cats	198	25	217	26	336	26
Rabbits	25	3	16	2	38	3
Other	113	15	120	5	212	7
Total	775	100	822	100	1279	100

**Fig. 1:**
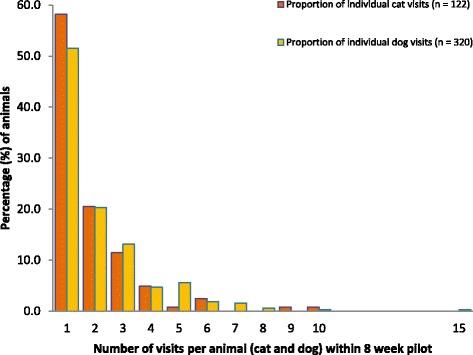
Visit frequency analysis for data extracted from a real veterinary practice over 8 weeks of data collection (Min visits 1, Max visits = 15)

Figure 1 presents data from visit frequency analysis using the extracted data. The analysis revealed 122 cats made a total of 299 visits to the practice during the first 8 week data collection period. Fifty nine percent of cats visited the veterinary practice only once, 21 % visited on two occasions, 12 % on three occasions and 5 % visited on four occasions up to a maximum of 10 visits. Three hundred and twenty dogs made a total of 683 visits to the veterinary practice during the same period. Fifty two percent visited the veterinary practice on only one occasion, 20 % on two occasions, 13 % on three occasions and 5 % on four occasions. A maximum of 15 repeat visits for one dog was recorded (Fig. 1).

Only six percent of visit records included a diagnostic code and 1 % included a VeNom installed code. The codes were mostly used by only one veterinarian and each VeNom code was combined with a practice specific diagnostic code.

Three veterinarians worked at the practice but additional members of the practice team (e.g. nursing staff and administrators) also added information into patient records during the two 8 week periods. In the first data collection period the analysis found the addition of information to the EPR by 14 different members of staff and similarly during the second data collection period 11 members of staff entered information. Overall there were a total of 15 staff members who added information into the PMS system during the data collection period with 10 people consistently involved across both data collection periods. Compliance within the practice was excellent and feedback from the practice was positive.

## Discussion

The method of extraction of patient records from both the test system and the real veterinary practice system using the XML Schema performed well, all targeted fields were extracted and all data within the targeted fields were extracted in full. All data excluded from the extraction method (e.g. billing, opt-out) were not present in the extracted dataset and all data was exported successfully and securely to the bespoke database for storage and analysis. The schema integrated well within the PMS and functionality was precise with little extra work required on the part of the veterinary team. An XML Schema was chosen for the project due to its flexibility to work across different computer systems. The XML schema had very little impact on the practitioners work load as it extracted the data that had already been recorded. Other methods of data extraction e.g. JSON are now available and may have advantages over XML, such as small file size. However the SPVS XML consortium and the PMS providers involved in that group were already familiar with and using XML schemas hence this method was the preferred method selected by collaborators in this project. The data extracted by the schema had an excellent level of precision. This work suggests that using an XML schema to extract clinical information from veterinary practice PMSs could provide the versatile approach needed for combining data from many practices utilising different PMS systems.

The data extraction highlighted the fact that the real veterinary practice added both VeNom codes and diagnostic codes to their PMS but only a few of the codes were used, with the veterinarians choosing general terms such as ‘consultation’ most frequently. Where a code was used, each VeNom code was combined with a practice specific diagnostic code resulting in an unnecessary duplication of information. In addition, terms were frequently duplicated due to synonymous use or differences in spelling which is also a common finding in human medicine where research reports little agreement between coded information and free text, even within the same record [24]. The vets at the practice also explained that the practice specific codes had been created by one veterinarian to assist with their own data input and it was not common practice for all veterinarians to record codes. This may explain their limited use within the practice. It is also possible veterinarians find it difficult to code a consultation if there is no definitive diagnosis at the time of the animal’s examination [27]. Many individuals within a practice may have a responsibility to update the EPR being used throughout the day. Overall for the research study presented here there were a total of 15 different staff members recorded. This multiple entry of data is unavoidable in a real working situation and it is important to consider when examining the data extracted. Additional issues with the extraction of patient data include the extraction of blank fields where information had not been entered by the veterinarian such as species information, and the inclusion of out of hours records or non-target species, e.g. farm species in this study. These issues do not compromise the data extracted but may contribute to workload increasing the time needed for analysis and data cleaning. It also highlights the need for continued feedback, communication and a close working relationship with the practice during the data collection period to ensure full compliance with the study protocol, such as the use of the opt-out function for out of hours and farm visits. A knowledge of the ways of working within the practice and how the veterinary team enter data is also very important for data quality.

## Strengths, limitations and future work

A potential barrier to practice involvement in clinical research is the time required to collect data. When it is straightforward to extract clinical record data and no extra data input is required of the practice, the time input required is minimal. Removing these potential barriers may improve practice compliance with research and using an XML schema to extract EPRs has the potential to address these barriers.

The data extracted can only be as good as that originally recorded. Data extracted from veterinary practice EPRs is unlikely to include standardised data as there is currently no standardised recording of information in veterinary medicine. Instead there may be spelling mistakes, shorthand, nuances and gaps in information which is to be expected from a busy working practice.

EPRs are increasingly being used for clinical research in veterinary medicine [9–12, 19, 20] and some work has begun to investigate methods to extract information collected during veterinary consultations from the EPR [20, 21]. However, to date no-one has published on a method used in the UK which could allow the extraction of veterinary patient data from a wide range of different PMS systems directly or on the completeness of the data extracted. The data extracted is often unstructured data, so to enable analysis, methods such as natural language processing or text mining would be required.

This manuscript describes a pilot study using a novel method of data extraction in one practice PMS. Prior to extending it to a large number of practices and PMSs, the accuracy and feasibility of the method needed to be established. This research group and their collaborators are now extending the work to more PMSs and more practices which provides some significant, but not unsurmountable, challenges. The download of data will need to be automated so that the staff of the practice do not need to be involved with the transfer of data. In addition data security measures, data quality checks and notification of failure of data transfer will also be required. The biggest challenge potentially will be the reluctance of PMS providers to engage in research that would make it easier for clients to move between systems. To overcome this challenge it will be necessary to work closely with the XML consortium, the profession, individual practices and the PMS providers to understand and address concerns and facilitate the use of this schema on a wider scale.

Criticism has been made of the use of data recorded in EPRs for practice-based research in the human healthcare field because it may be incomplete [22, 23] or because its completeness has not been validated [22–25]. Additionally there has been concern over data sharing and confidentiality [26] which may affect participation by the veterinary profession. Professional issues may also need to be addressed such as the concern over anonymity and confidential ways of working for both clients and vets. This includes concern over the regulation of prescribing drugs, the veterinary prescription ‘cascade’, and disclosure of veterinary identity.

Although there are some obstacles to overcome, this method has been shown to be highly successful and novel with great value to veterinary epidemiology and practice-based research.

## Conclusion

The extraction method proved to be reliable and efficient and did not interfere with clinical practice. The data extracted yielded a great deal of information for analysis and practice-based research. The potential value to the veterinary profession and the opportunity for research is sizeable as the type of PMS used by a practice is not a barrier for involvement in research that uses this method. The results of this group of studies, the success of the methodology and the high level of precision for the extraction system, provide great encouragement for the future of practice-based research utilising medical informatics and XML language technology.

## Additional file

Additional file 1: Clinical Evidence Schema as text file. (TXT 3 kb)

## Abbreviations

CE: Clinical evidence
EPR: Electronic patient record
JJD: Julie Jones-Diette
PMS: Practice management software
SPVS: Society of practising veterinary surgeons
XML: Extensible mark-up language
